# Mitochondrial Dysfunction in Down Syndrome associated Alzheimer’s Disease

**DOI:** 10.1002/alz.091641

**Published:** 2025-01-03

**Authors:** Keith P Smith, Taylor A. Strope, Brittany M Hauger, Colton R Lysaker, Cynthia Shaddy‐Gouvion, Mohammad Haeri, Elizabeth Head, Russell H Swerdlow, Heather M Wilkins

**Affiliations:** ^1^ 3901 rainbow blvd, Kansas City, KS USA; ^2^ University of Kansas Medical Center, Kansas City, KS USA; ^3^ The UC Irvine Institute for Memory Impairments and Neurological Disorders (UCI MIND), Irvine, CA USA; ^4^ University of Kansas Alzheimer’s Disease Research Center, Fairway, KS USA

## Abstract

**Background:**

Mitochondrial dysfunction and Aβ accumulation are hallmarks of Alzheimer’s disease (AD). However, the role of these pathologies in Down Syndrome associated Alzheimer’s Disease (DSAD) is unknown. Decades of research describe a relationship between mitochondrial function and Aβ production. Amyloid precursor protein (APP), from which Aβ is generated, is found in mitochondria. APP and Aβ alter mitochondrial function, while mitochondrial function alters Aβ production from APP. How these interactions contribute to DSAD pathology and progression are unknown. Here we interrogated the association of full‐length APP with mitochondria, mitochondrial function, and AD pathological hallmarks.

**Method:**

ND (n = 10, without DS) and DS associated Alzheimer’s Disease (DSAD, n = 10) postmortem brain tissue was obtained from the University of California Irvine. A human iPSC line was purchased from WiCell with Trisomy 21 and a isogenic control line which underwent Crispr/Cas9 genome editing to remove the extra chromosome 21 copy. iPSC models were differentiated into neurons, astrocytes, and cerebral organoids using StemCell Technologies reagents and protocols. We examined mitochondrial function using a Seahorse XF analyzer. We measured full‐length APP protein levels in whole cell extracts and mitochondrial fractions via Western Blotting. We measured Aβ levels with ELISA kits from ThermoFisher.

**Result:**

DSAD postmortem brain tissue had reduced mitochondrial function regardless of sex. Full‐length APP levels were significantly higher in mitochondrial fractions in DSAD brain tissue. Full‐length APP levels in mitochondrial fractions correlated with mitochondrial function. Higher mitochondrial APP (full‐length) levels associated with lower mitochondrial function. iPSC derived models showed similar phenotypes to postmortem brain tissues, including increased mitochondrial APP levels, and decreased mitochondrial function.

**Conclusion:**

We describe a relationship between mitochondrial APP accumulation, and mitochondrial function. These data support a centralized role for mitochondrial function in APP physiology and APP may play a role in modulating mitochondrial function. Further, DSAD postmortem tissue and iPSC models show significant mitochondrial dysfunction.

